# Co-occurrence of opisthorchiasis and diabetes exacerbates morbidity of the hepatobiliary tract disease

**DOI:** 10.1371/journal.pntd.0006611

**Published:** 2018-06-28

**Authors:** Apisit Chaidee, Sudarat Onsurathum, Kitti Intuyod, Patchareewan Pannangpetch, Chatlert Pongchaiyakul, Porntip Pinlaor, Chawalit Pairojkul, Wannaporn Ittiprasert, Christina J. Cochran, Victoria H. Mann, Paul J. Brindley, Somchai Pinlaor

**Affiliations:** 1 Department of Parasitology, Faculty of Medicine, Khon Kaen University, Khon Kaen, Thailand; 2 Cholangiocarcinoma Research Institute, Khon Kaen University, Khon Kaen, Thailand; 3 Department of Pharmacology, Faculty of Medicine, Khon Kaen University, Khon Kaen, Thailand; 4 Department of Medicine, Faculty of Medicine, Khon Kaen University, Khon Kaen, Thailand; 5 Centre for Research and Development of Medical Diagnostic Laboratories, Faculty of Associated Medical Sciences, Khon Kaen University, Khon Kaen, Thailand; 6 Department of Pathology, Faculty of Medicine, Khon Kaen University, Khon Kaen, Thailand; 7 Department of Microbiology, Immunology and Tropical Medicine, and Research Center for Neglected Diseases of Poverty, School of Medicine & Health Sciences, The George Washington University, Washington, D.C., United States of America; University of Queensland, AUSTRALIA

## Abstract

Complications arising from infection with the carcinogenic liver fluke *Opisthorchis viverrini* cause substantial morbidity and mortality in Thailand and adjacent lower Mekong countries. In parallel, the incidence rate of diabetes mellitus (DM) is increasing in this same region, and indeed worldwide. Many residents in opisthorchiasis-endemic regions also exhibit DM, but the hepatobiliary disease arising during the co-occurrence of these two conditions remains to be characterized. Here, the histopathological profile during co-occurrence of opisthorchiasis and DM was investigated in a rodent model of human opisthorchiasis in which diabetes was induced with streptozotocin. The effects of excretory/secretory products from the liver fluke, *O*. *viverrini* (OVES) on hepatocyte and cholangiocyte responses during hyperglycemic conditions also were monitored. Both the liver fluke-infected hamsters (OV group) and hamsters with DM lost weight compared to control hamsters. Weight loss was even more marked in the hamsters with both opisthorchiasis and DM (OD group). Hypertrophy of hepatocytes, altered biliary canaliculi, and biliary hyperplasia were more prominent in the OD group, compared with OV and DM groups. Profound oxidative DNA damage, evidenced by 8-oxo-2'-deoxyguanosine, proliferating cell nuclear antigen, and periductal fibrosis characterized the OD compared to OV and DM hamsters. Upregulation of expression of cytokines in response to infection and impairment of the pathway for insulin receptor substrate (IRS)/phosphatidylinositol-3-kinases (PI3K)/protein kinase B (AKT) signaling attended these changes. *In vitro*, OVES and glucose provoked time- and dose-dependent effects on the proliferation of both hepatocytes and cholangiocytes. In overview, the co-occurrence of opisthorchiasis and diabetes exacerbated pathophysiological damage to the hepatobiliary tract. We speculate that opisthorchiasis and diabetes together aggravate hepatobiliary pathogenesis through an IRS/PI3K/AKT-independent pathway.

## Author summary

Infection with the food-borne liver fluke *Opisthorchis viverrini* frequently causes bile duct cancer, a prominent public health concern in the greater Mekong sub-region countries. In like fashion, diabetes mellitus is a major public health problem in this region and worldwide. The interrelationships of these two conditions were investigated in hamsters and in tissue cultures of human liver cells. Liver fluke infection exacerbated diabetes-related disease in the liver and bile ducts in hamsters and in the cultured cells. The findings of this investigation revealed that infection with liver fluke parasites during diabetes leads to more serious disease in the liver than is due to either condition alone.

## Introduction

More than 45 million people are infected with fish-borne liver flukes, trematodes of the family Opisthorchiidae [1], primarily species of the genera *Opisthorchis* and *Clonorchis*. Numerous residents of regions where undercooked fish harboring opisthorchiid metacercariae are typically consumed suffer from these conditions, particularly in endemic sites in Southeast Asia and Eurasia including western Siberia. Among the causative species, *Opisthorchis viverrini* is the major problem in the lower Mekong River basin, and its prevalence is highest in northeastern Thailand where > 6 million people are infected [2]. Opisthorchiasis causes injury by mechanical and immunopathological processes, leading to cholangitis and periductal fibrosis [3]. Infection with *O*. *viverrini* is strongly associated with development of cholangiocarcinoma (CCA) and hence infection with this biliary tract pathogen has been classified as a group 1 carcinogen by the International Agency for Research on Cancer [4]. Northeastern Thailand has the highest incidence of intrahepatic CCA in the world; >100 and 50 cases per 100,000 person-years in men and women, respectively [5–7].

Earlier reports using rodent models revealed that during the acute stage of infection, opisthorchiasis provokes histopathological changes including inflammation of the second order bile ducts and portal connective tissues. After the flukes mature, the infection induces hyperplasia, adenomatous formation and granulomatous responses to the worms and their eggs, leading to biliary stasis, periductal fibrosis and portal scaring [8]. During chronic infection, apoptosis of damaged biliary cells is inhibited, biliary epithelia cells proliferate and expression of pro-inflammatory cytokines is upregulated. In addition, aberrant development of bile canaliculi takes place [9, 10]. Antigens released from the worms translocate to the cholangiocytes, to hepatocytes, and to intra-hepatocyte gaps [11]. Although the role of these antigens on cholangiocytes has been partially dissected, the effects of liver fluke metabolites on hepatocytes have not yet been established.

As with CCA [6], the worldwide prevalence of diabetes mellitus (DM) is increasing, especially in the developing world [12] including in Thailand [13]. Among its diverse sequelae, DM increases risk for bacterial and fungal infections [14]. The influence of concurrent infection with helminth parasites during DM remains controversial [15, 16]. Deciphering the underlying mechanisms of the interactions between infection with helminths and DM will enhance our understanding of the consequences of comorbidity of diabetes during infection [17]. Clinical and experimental observations reveal that DM-induced hepatobiliary disease arises from immunopathological processes, hepatocyte damage and biliary hyperplasia [18, 19]. Carbonyl stress also has been implicated in hepatic tissue during opisthorchiasis [20, 21].

Chronic opisthorchiasis provokes immunopathological processes that lead to biliary periductal fibrosis and other hepatobiliary lesions [3]. As with helminth infection in general, elevated Th1-inducing cytokines dominate at the acute phase of opisthorchiasis whereas during the chronic stage, the expression of Th2-inducing cytokines becomes prominent [22]. However, during DM, cytokine dynamics following infection may be altered. Although the influence of helminth infection on the suppression of diabetes is established in relation to peripheral blood glycemia and the pancreas, effects in the liver and other sites are less clear [23, 24]. We hypothesized that infection with *O*. *viverrini* during DM exacerbates hepatobiliary disease compared to DM in the absence of infection. To address the hypothesis, we investigated the pathophysiology of the hepatobiliary tract following infection with *O*. *viverrini* in hamsters during streptozotocin-induced Type 1 diabetes (T1D). The effects of excretory/secretory products from the liver fluke on both hepatocytes and cholangiocytes under glycemic conditions also were investigated.

## Results

### Opisthorchiasis during diabetes exacerbates morbidity of hepatobiliary tract disease

**Fig 1:**
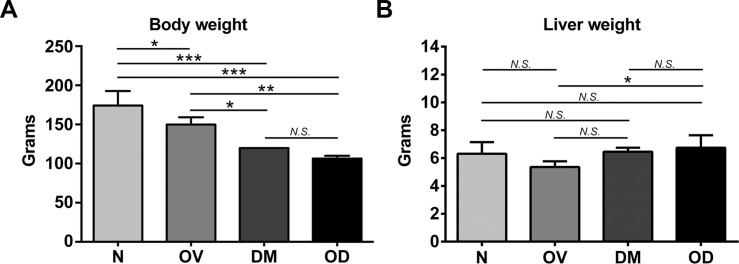
Whole body and liver weights of hamsters during infection with liver flukes and diabetes. Panel A, body weight; B, liver (wet) weight. Thirty-two hamsters were included in four treatment groups: group N, control normal hamsters; OV, infected with *Opisthorchis viverrini*; DM, diabetic; and OD, both diabetic and infected with *O*. *viverrini*. In the DM group, diabetes was induced by intraperitoneal injection of 50 mg/kg body weight of streptozotocin (STZ). Type 1 diabetes (T1D) hamster was evaluated by glucometer at two weeks post-STZ injection. Fasting blood glucose level (FBG) was estimated in all animals and those with FBG ≥ 250 mg/dL were enrolled into the DM or OD groups. In OD, T1D hamsters were infected with 50 metacercariae of *Opisthorchis viverrini* and euthanized one month after infection. Findings are presented as mean ± S.D.; *P ≤ 0.05; **P ≤ 0.01; and ***P ≤ 0.001; *N*.*S*., not significant.

**Fig 2:**
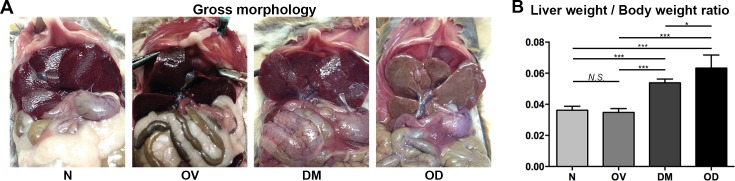
**Gross hepatobiliary tract disease (panel A) and liver weight/ body weight ratio (B) among hamsters following infection with *Opisthorchis viverrini* and induction of diabetes.** The study included four treatment groups of hamsters, as in Fig 1.

**Fig 3:**
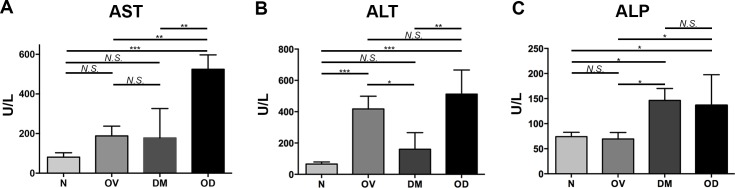
Liver function enzyme levels. Panel A, **s**erum AST; B, ALT; C, ALP following infection with the liver fluke *Opisthorchis viverrini* and induction of Type 1 diabetes. AST, ALT and ALP were assessed by automated spectrophotometer. Treatment groups and statistical symbols as in Fig 1.

Four groups of hamsters were studied; these were denoted as groups N, control (normal) hamsters; OV, infected with *Opisthorchis viverrini*; DM, diabetic; and OD, both diabetic and infected with *O*. *viverrini*. T1D was induced with streptozotocin (STZ) after which the OD hamsters were infected *O*. *viverrini*. The rodents were euthanized one month after infection. Whereas infection significantly reduced fasting blood glucose from 365.30 ± 73.23 mg/dL to 263.40 ± 120.75 mg/dL (*P* ≤ 0.05), in similar fashion to reports involving with other helminth infections [25], hyperglycemia was maintained throughout the remainder of the investigation. The gross appearance of the liver was discolored markedly yellowish and the liver to whole body weight ratio increased in OD (Figs 1 and 2). In comparison with N and with OV, diabetic hamsters (DM) exhibited hepatic jaundice and hepatomegaly. Elevated levels of AST, ALT, and ALP were recorded in OD, indicating altered liver function that, in turn, likely resulted from the joint insults of infection and diabetes to the hepatobiliary tissues (Fig 3). These disease manifestations indicated that opisthorchiasis during diabetes exacerbated hepatobiliary disease in comparison to the morbidity of liver fluke infection or diabetes.

### Liver fluke infection and diabetes additively increase severity of hepatocyte damage

**Fig 4:**
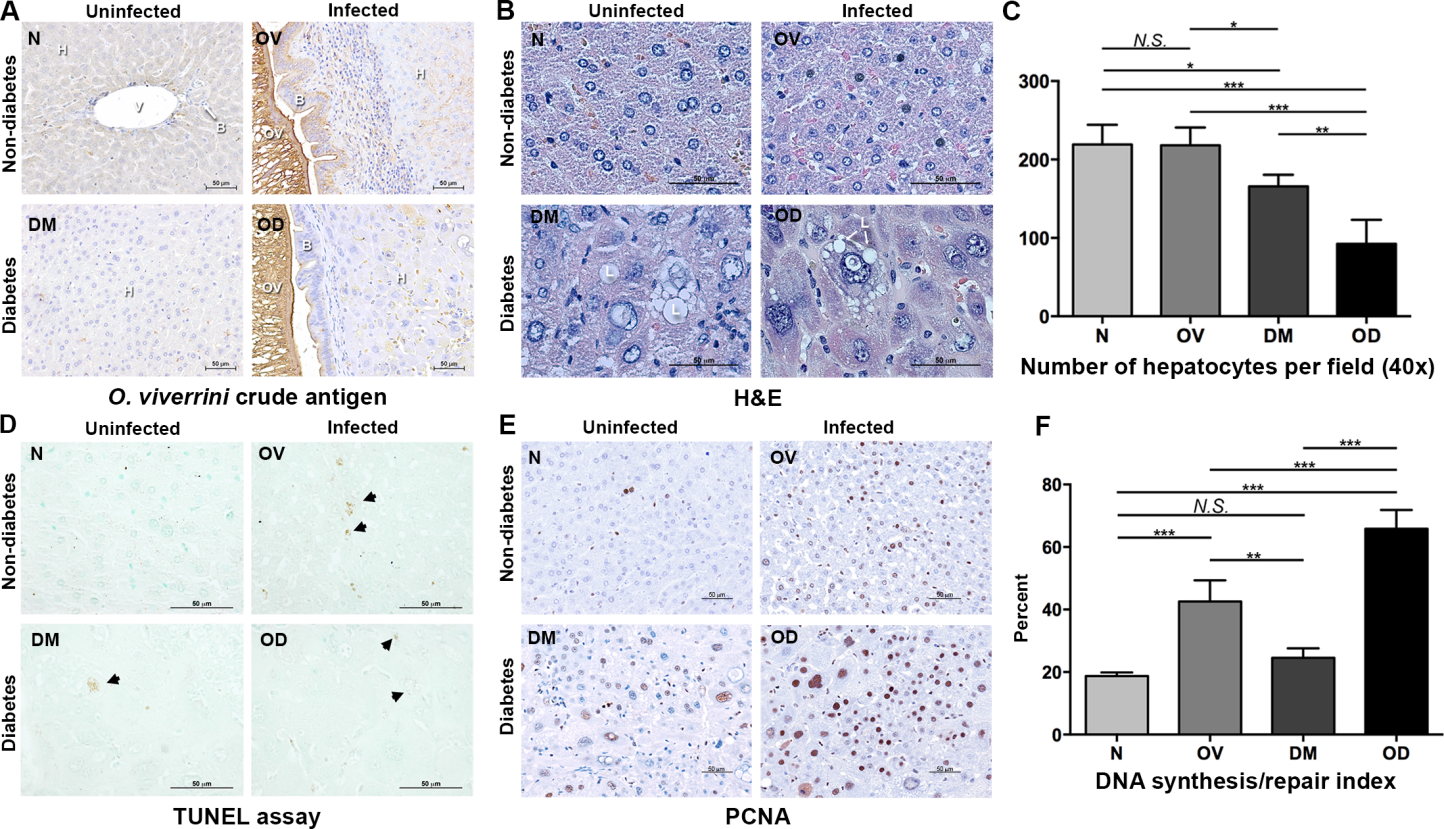
Histopathological changes during concurrent opisthorchiasis and diabetes. Thin sections of liver were stained with antibodies to detect antigens of *Opisthorchis viverrini* or PCNA. Panel A, Thin sections of liver immunostained for *O*. *viverrini* antigen; B, stained with hematoxylin and eosin (H &E); C, numbers of hepatocytes; D, TUNEL assay (x400 magnification); the arrowheads indicate positive cells; E, immunohistochemical assay for PCNA; F, numbers of PCNA-positive hepatocytes per field of each of 10 sites per sample (under x200 original magnification). V = portal vein; H = hepatocyte; B = bile duct; OV = *O*. *viverrini*; L = lipid droplet. Treatment groups as in Fig 1.

Thin sections of liver were immuno-stained with antiserum specific for *O*. *viverrini* antigens. The presence and profile of *O*. *viverrini* antigens were observed in bile duct epithelium and sites adjacent to the liver flukes and also, notably, within hepatocytes of hamsters in OV and OD groups (Fig 4A). Hypertrophy of hepatocytes was observed in hamsters with diabetes, in both DM and OD (Fig 4B) whereas this was not seen in OV and N. When compared to N and OV, the number of hepatocytes per high power field was significantly lower in DM and lowest in OD (Fig 4B and 4C), indicating that the hepatocellular hypertrophy was prominent during concurrent opisthorchiasis and diabetes.

Given the elevated levels of liver enzymes in OD hamsters, we examined the extent of liver injury and the compensatory response. By TUNEL assay, a method to monitor ongoing apoptosis (detected as DNA fragmentation labeled by terminal deoxynucleotidyl transferase enzyme and brominated nucleotide, BrdU), DNA fragmentation was detected during both diabetes and/or *O*. *viverrini* infection with the similar levels in OV, DM and OD (Fig 4D). DNA replication and DNA repair, as indicated by the expression of proliferating cell nuclear antigen (PCNA), significantly increased during infection (OV) and diabetes (DM) compared to the control hamsters. Among all groups, OD exhibited most PNCA-positive nuclei (Fig 4E and 4F), indicating that, in addition to hepatocyte hypertrophy, infection with liver flukes during diabetes provoked hepatocyte proliferation.

### Liver fluke infection during diabetes aggravates biliary tract morbidity

**Fig 5:**
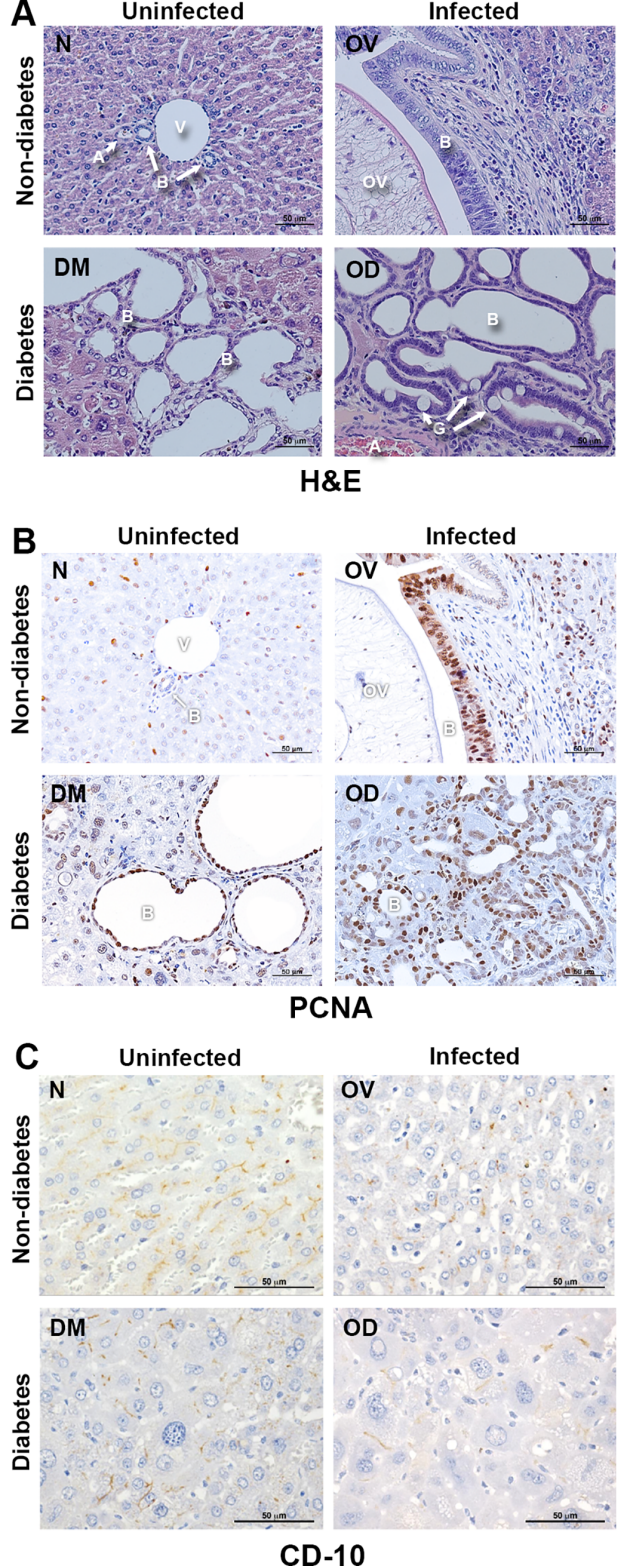
Histopathological changes in the biliary tract during concurrent opisthorchiasis and diabetes. Panel A, sections stained with hematoxylin and eosin (H&E); B, immunohistochemical analysis for proliferating cell nuclear antigen, PCNA (under x200 original magnification); C, stained for CD10, a marker of bile canaliculi (under x400 original magnification). V = portal vein; H = hepatocyte; B = bile duct; OV = *O*. *viverrini*; A = artery; G = goblet cell. Symbols for treatment groups as in Fig 1.

Prominent infiltration of inflammatory cells, accumulation of fibrosis, and proliferation of biliary epithelia were similarly seen with the previously reported in *O*. *viverrini*-infected hamsters [26]. Here, proliferation of the small bile ducts also was evident in DM and marked in OD hamsters. Goblet cell metaplasia had established in OD (Fig 5A), indicating that the infection induced severe biliary duct injury in the setting of diabetes. In addition, livers of the OD hamsters underwent the most intense cellular proliferation of biliary epithelium, as evidenced by staining for PCNA (Fig 5B). PCNA scores, which graded numbers of PCNA-positive nuclei in bile ducts, reflected this comparative profile among the groups (Table 1).

**Table 1:** PCNA-positive pattern in biliary tract, the grade of pattern of bile canaliculi, grade of fibrosis and parentage of 8-oxo-dG-positive cells.

Treatment	PCNA-positive pattern (mean ± S.D.)	CD-10 pattern (mean ± S.D.)	Fibrosis grade (mean ± S.D.)	8-oxo-dG grade (mean ± S.D.)
Normal	1 ± 0.577	2.76 ± 0.476	0.33 ± 0.272	2.08 ± 2.080
*O*. *viverrini* infected	2.25 ± 0.829*	1.95 ± 0.749***	1.67 ± 0.577*	9.73 ± 5.180
Diabetes	1.33 ± 0.471^†^	1.63 ± 0.718***^,^ ^†^	0.83 ± 0.236^†^	3.40 ± 4.327
*O*. *viverrini* and diabetes	2.82 ± 0.385***^,^ ^†^^,^ ^‡‡^	1.20 ± 0.723***^,^ ^†††^^,^ ^‡^	2.50 ± 0.236***^,^ ^†^^,^ ^‡‡^	69.05 ± 14.617***^,^ ^†††^^,^ ^‡‡^

* *P* ≤ 0.05 when compared to normal (N)

*** *P* ≤ 0.001 when compared to N

^†^ *P* ≤ 0.05 when compared to *O*. *viverrini*-infected (OV)

^†††^ *P* ≤ 0.001 when compared to OV

^‡^ *P* ≤ 0.05 when compared to diabetes (DM)

^‡‡^ *P* ≤ 0.01 when compared to DM

Cluster of differentiation 10 (CD10), a neutral metalloendopeptidase, is located in microvilli of the canalicular membrane of hepatocytes and well-known marker for monitoring the function and disease of bile canaliculi [27, 28]. Immunostaining for CD10 was employed to assess the impact on bile canaliculi of concurrent opisthorchiasis and diabetes. CD10 was substantially depleted in OV, DM, and OD compared to the control hamsters (N) (Fig 5C), reflecting aberrant profiles of the canaliculi. Notably, OD hamsters displayed the most irregular profile (Table 1).

### Co-occurrence of opisthorchiasis and diabetes additively exacerbates hepatic fibrosis and oxidative DNA damage

**Fig 6:**
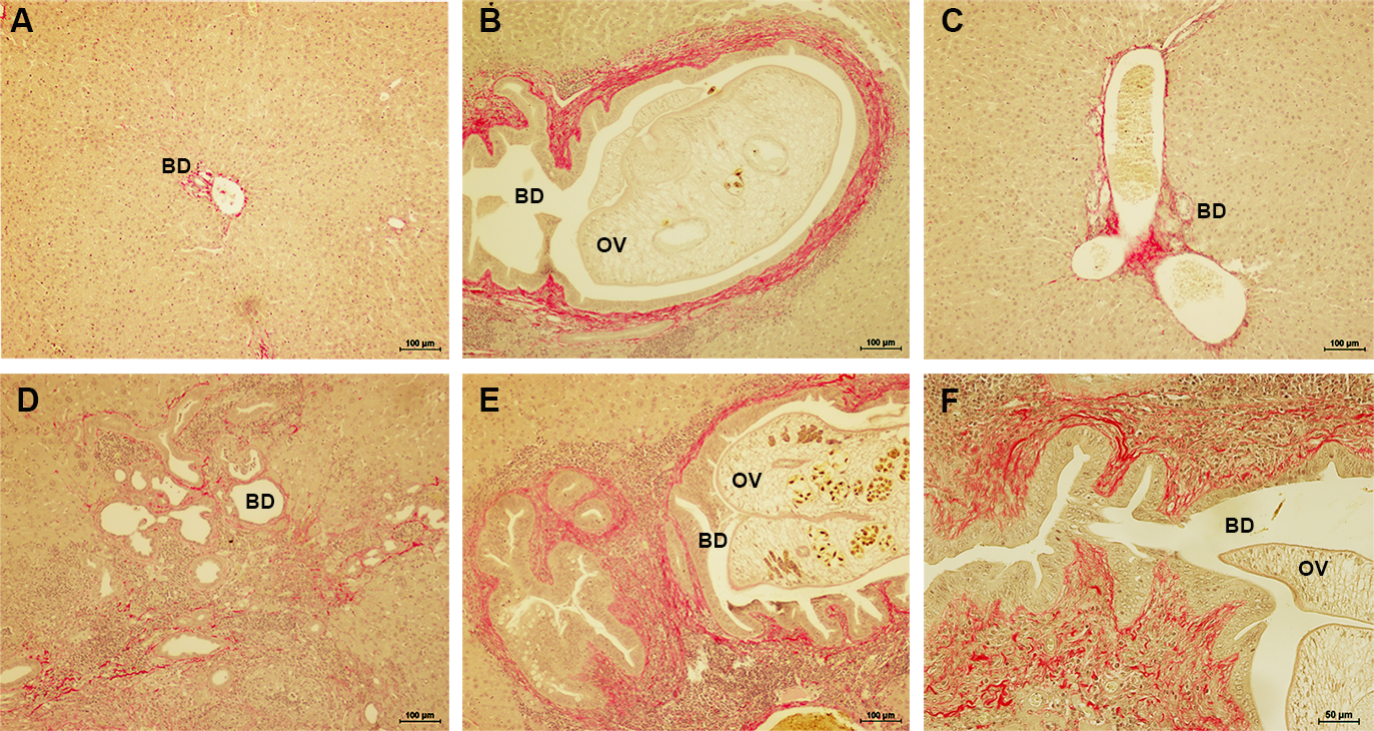
Hepatic fibrosis in liver of hamsters. Appearance of fibrotic tissue in the liver of normal, *O*. *viverrini*-infected, diabetic hamsters and the concurrent of opisthorchiasis and diabetes was evaluated by staining with Picrosirius red (PSR). Positive PSR staining of fibrosis is shown as bright red (under x100 and x200 original magnifications). Panel A, treatment group N; B, group OV; C, group DM; D–F, group OD group, with (E) small and (F) large bile ducts containing a liver fluke. OV = *O*. *viverrini*, BD = Bile duct. Treatment groups as in Fig 1.

Since periductal fibrosis is the key histopathological finding and is known to increase during chronic infection with *O*. *viverrini* [26], we investigated the effect of infection and/or diabetes on periductal fibrosis by staining the thin sections of liver with Picrosirius red (PSR), which stains collagen types I and III in paraffin-embedded tissue sections. Compared to normal liver (Fig 6A), fibrosis (as established by positive staining with PSR) surrounding liver flukes with the lumen of larger bile ducts was prominent in the OV group hamsters (Fig 6B). A modest increase (compared to N) in occurrence of fibrotic lesions was evident in the portal triad and small bile ducts in DM hamsters (Fig 6C). In OD, marked periductal fibrosis was present both surrounding liver flukes in larger diameter bile ducts and also surrounding the smaller, second-order ducts (Fig 6D, 6E and 6F, Table 1).

**Fig 7:**
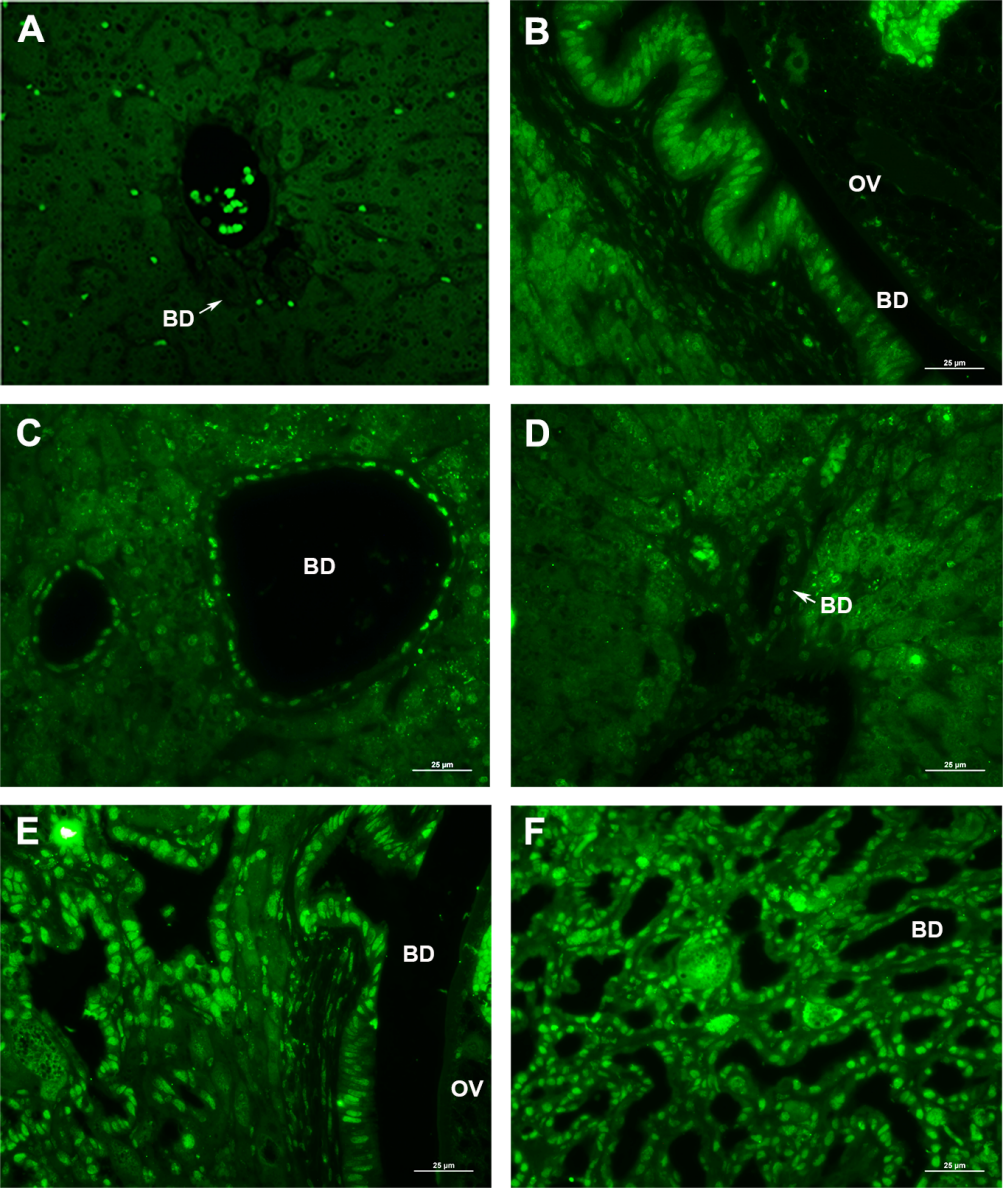
Oxidative DNA damage in hamster liver. Formation of 8-oxo-dG in the liver of *O*. *viverrini*-infected, diabetic hamsters evaluated by immunofluorescence staining. Representative micrographs showing nuclei stained bright green, to reveal 8-oxo-dG (x400 magnification). Panel A, N group; B, OV group; C and D, DM group of large and small bile ducts; E, F, OD group of large bile duct with parasite and small bile ducts. OV = *O*. *viverrini*, BD = Bile duct. Treatment groups as in Fig 1.

Oxidative stress caused by either opisthorchiasis or diabetes shares connections to disease outcome [18, 29]. Staining was also undertaken for 8-oxo-dG, a hallmark of the oxidative DNA damage. Livers of the control hamsters exhibited background immunohistofluorescence signals for 8-oxo-dG (2.08 ± 2.080; Fig 7A, Table 1). Faint to modest positive of 8-oxo-dG staining was seen in the DM hamsters (3.40 ± 4.327; Fig 7C and 7D, Table 1). By contrast, during infection with *O*. *viverrini*, there was increased 8-oxo-dG staining in nuclei of biliary duct epithelial cells and in inflammatory cells in the proximal periductal tissue (9.73 ± 5.180; Fig 7B, Table 1). Moreover, the level 8-oxo-dG staining was massively elevated in these same regions in the OD group (69.05 ± 14.617; Fig 7E and 7F, Table 1), demonstrative of synergy in oxidative DNA damage from the concurrent infection and diabetes. In addition, OV and OD displayed divergent staining patterns. In OV and DM, the positive signals were evident mainly in the larger and smaller bile ducts, respectively. In OD, the intense 8-oxo-dG signals were present in the larger and smaller bile ducts and in the hepatocytes.

### Overexpression of Th1/Th2 cytokines during concurrent opisthorchiasis and diabetes

**Fig 8:**
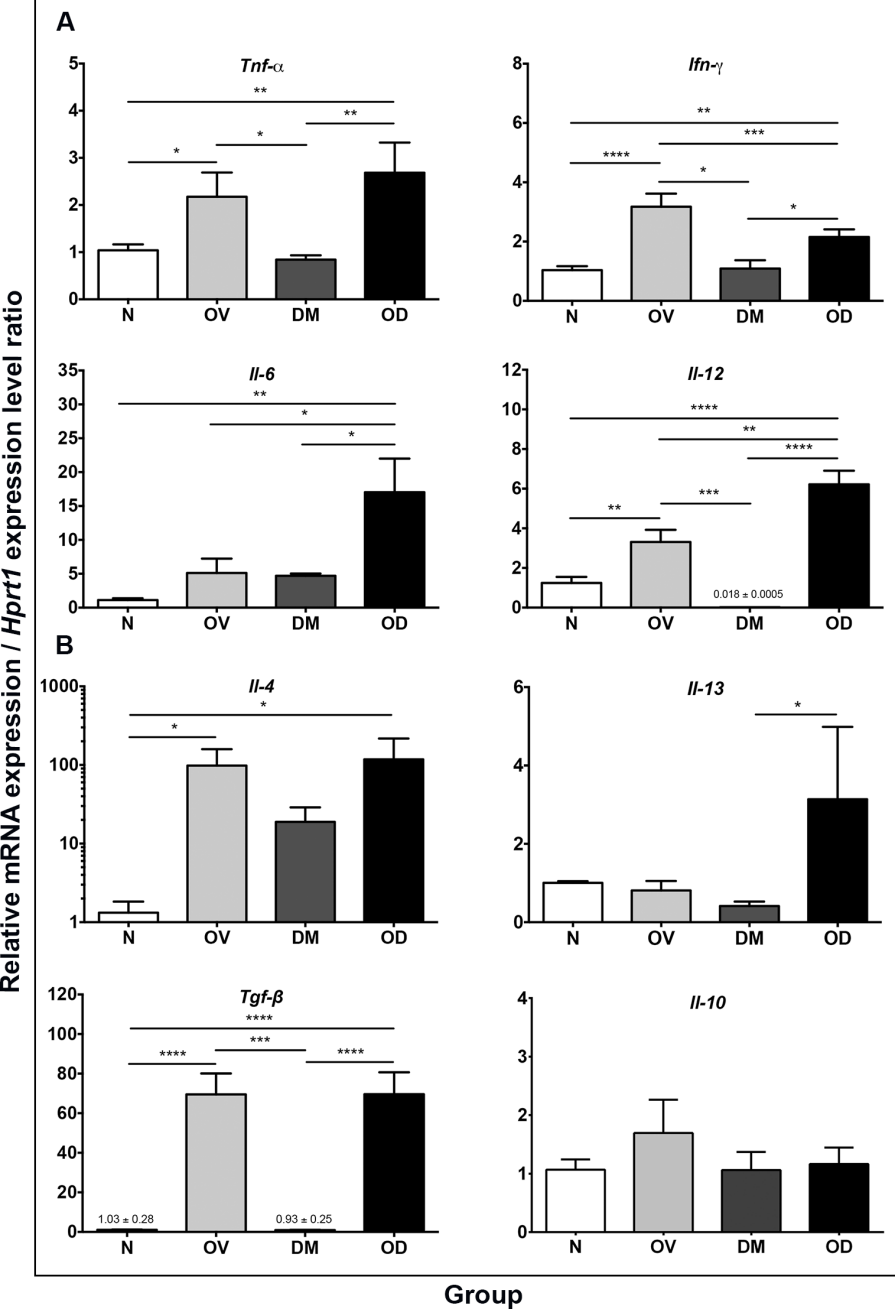
Expression of infection-related cytokines. Panel A, the expression of cytokine mRNA during the acute and, B, chronic phases of opisthorchiasis were investigated using real-time PCR. The findings are presented as fold change over signal in the normal hamsters, following normalizing against the expression level of the reference gene, *hprt1*. Symbols as for Fig 1.

The action of cytokines in the setting of opisthorchiasis and/or diabetes may contribute to severity of hepatobiliary disease. Strength of immune response in hepatic tissues was assessed indirectly by establishing cytokine mRNA expression levels by quantitative reverse transcription polymerase chain reaction (qRT-PCR). Two groups of cytokines, Th1-related (*Tnf-α*, *Ifn-γ*, *Il-6*, and *Il-12*) and Th2-related (*Il-4*, *Il-13*, *Tgf-β*, and *Il-10*) cytokines were measured and the findings presented in Fig 8A and 8B, respectively. Transcription levels of *Tnf-α*, *Ifn-γ*, *Il-12*, *Il-4*, *and Tgf-β* increased significantly in the OV and OD groups, compared to N. Expression of *Il-6* significantly increased in OD group; there also was a modest increase in OV, but not significantly. Expression of *Il-13* significantly increased in OD, whereas mRNA expression of *Il-10* significantly increased in OV. Except for *Il-4*, differences were not apparent between DM and N.

### Impaired IRS/PI3K/AKT signaling during concurrent diabetes and opisthorchiasis

**Fig 9:**
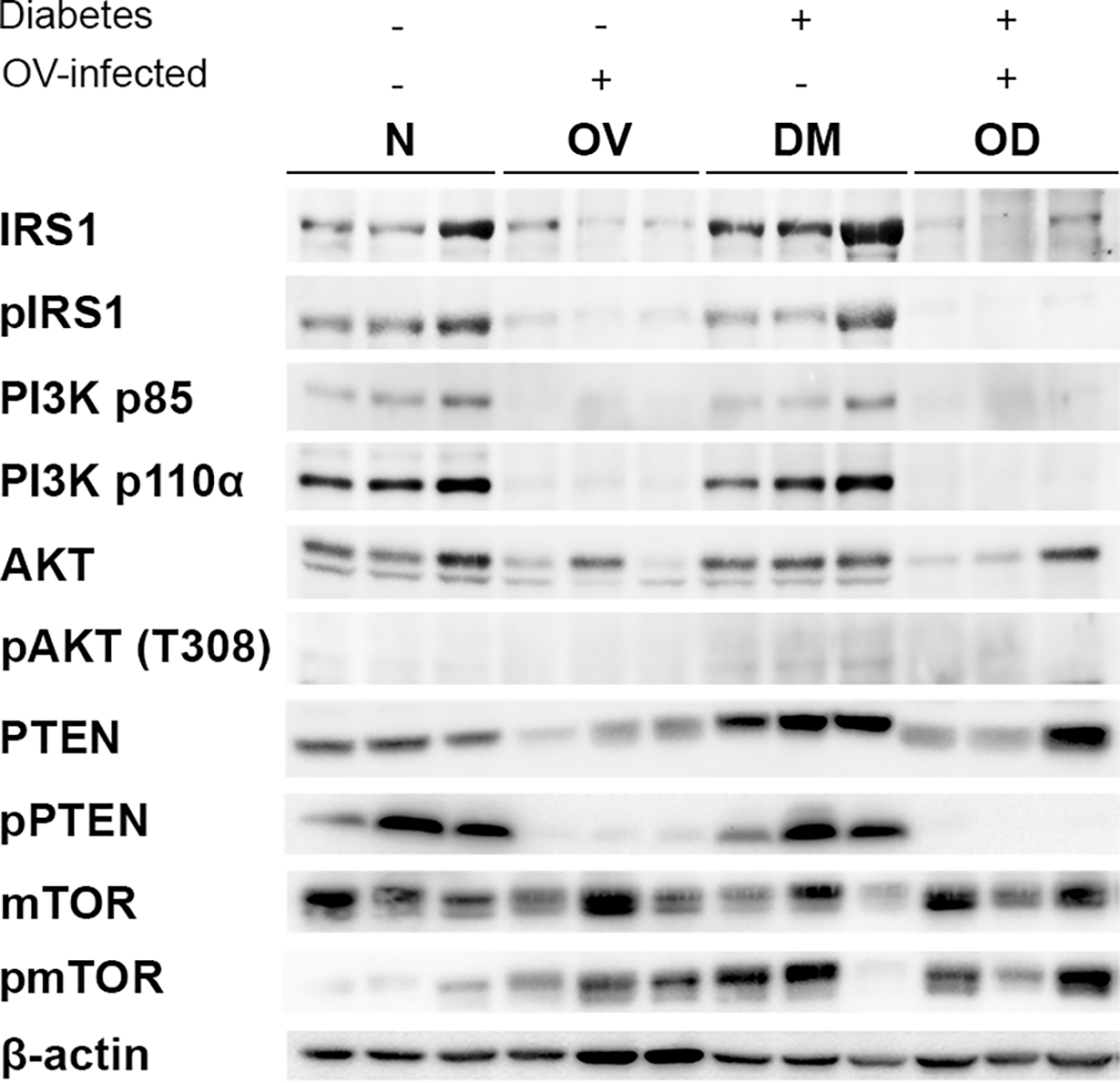
Expression of IRS/PI3K/AKT signaling during diabetes and liver fluke infection using western blotting. Findings presented by group for the total and phosphorylated forms of pathway proteins; signals presented from each of three livers from representative hamsters, with duplicate independent, experiments. Treatment groups N, OV, DM and OD, as in the legend to Fig 1.

To gain insights into the involvement of the PI3K/AKT/mTOR pathway on proliferation of hepatocytes and cholangiocytes, expression levels of principal components of the pathway(s) were evaluated in comparative western blots (Fig 9). Levels of IRS1, PI3K, AKT, and their phosphorylated forms markedly increased during diabetes, in the DM group. By contrast, expression levels of these proteins decreased markedly in the OV and OD group hamsters, when compared to N. Moreover, a similar trend obtained with PTEN, a negative regulator of PI3K/AKT, and its phosphorylated form. Of note, a trend towards increased mTOR protein expression was not apparent in OV, DM and OD, although the level of the phosphorylated form increased.

### Response of hepatocytes and cholangiocytes to liver fluke antigens and glucose

**Fig 10:**
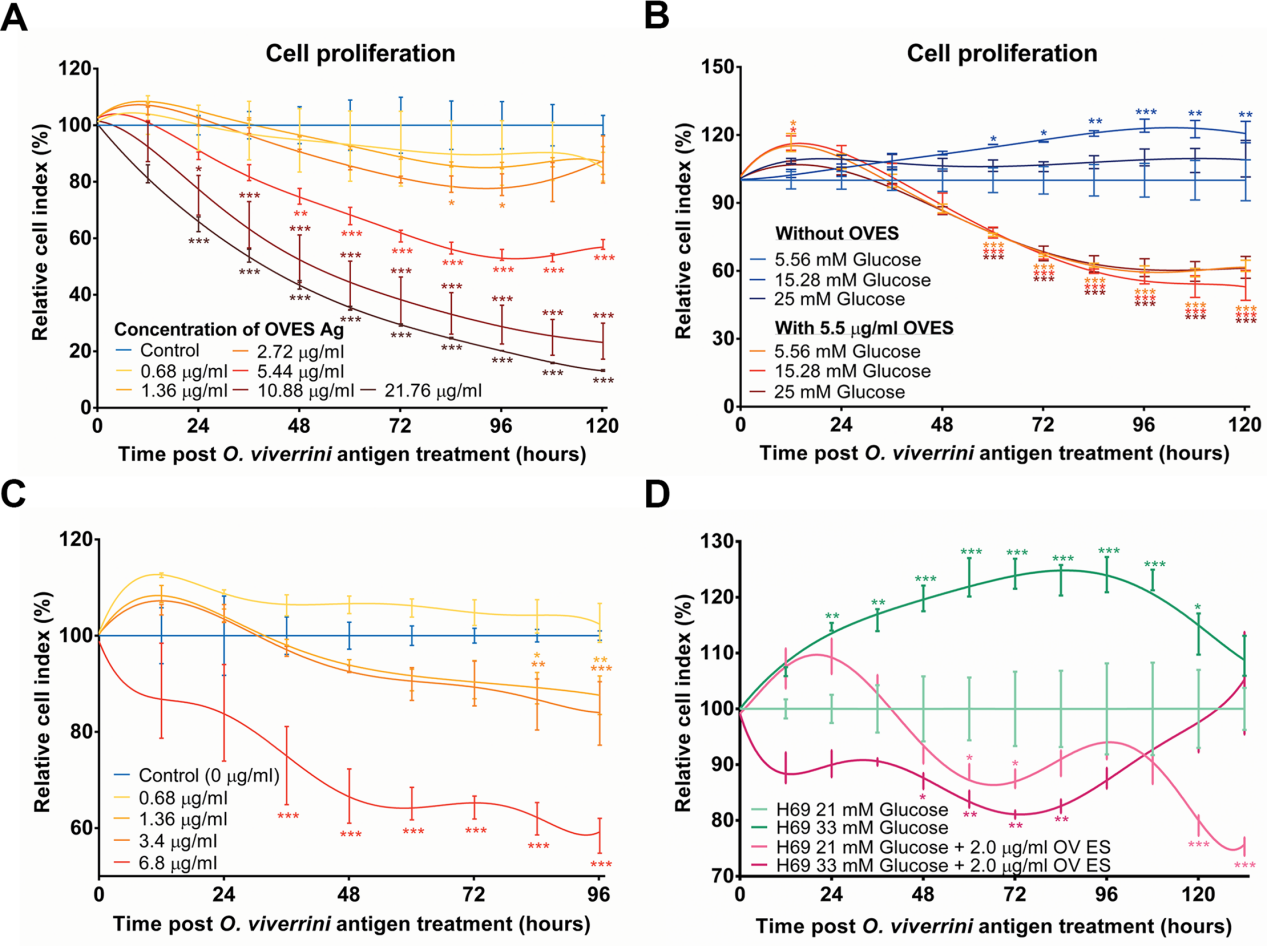
Impact of excretory/secretory products of *Opisthorchis viverrini* (OVES) and glucose concentration on growth and proliferation of hepatocytes (hepatoma cell line, HepG2) and cholangiocytes (H69). Growth of HepG2 (panels A, B) and H69 (C, D) cells was monitored in real time using the xCELLigence DP-Real-Time Cell Analysis (RTCA) system, with growth curves displayed using normalized cell index values. Data are presented as mean ± S.D; *P ≤ 0.05; **P ≤ 0.01; and ***P ≤ 0.001 compared to controls.

Following up on the findings of concurrent opisthorchiasis and diabetes in the hamsters on hepatocellular hypertrophy, proliferative effects of OVES and glucose on hepatocytes and cholangiocyte proliferation were investigated using the HepG2 (Fig 10A and 10B) and H69 cell (Fig 10C and 10D) lines. Cell growth was monitored in real time, using the xCELLigence system, in glucose at increasing concentration and with OVES. HepG2 cells were incubated in increasing glucose and exposed to 1.36–21.76 μg/mL OVES. OVES progressively decelerated the proliferation of HepG2s in a dose-dependent manner (Fig 10A). Whereas glucose at 15.28 mM stimulated increased proliferation of HepG2, higher concentration of glucose led to reduction in rate of proliferation. Moreover, 5.5 μg/mL OVES continued to inhibit cell growth, including at 15.28 mM glucose (Fig 10B). With the cholangiocyte line H69, OVES inhibited cellular proliferation generally in a dose-dependent manner (Fig 10C). When these cells were maintained at hyperglycemic condition (33 mM glucose), they proliferated faster than in normal (21 mM) glucose (Fig 10D). Notably, in 2.0 μg/mL OVES (low dose) proliferation of H69s increased through the first 48 h in 33 mM glucose; subsequently, growth was inhibited. Notable also was that 33 mM glucose, OVES at 2.0 μg/mL represented the only regimen where the cholangiocytes continued to proliferate at 96 hours; cell populations in the treatment groups had crashed or died by that time (Fig 10D).

## Discussion

The diabetic liver and the non-diabetic liver during opisthorchiasis share some similar disease features and, accordingly, we hypothesized that the coincidence of liver fluke infection and diabetes would exacerbate the morbidity, in similar fashion to the situation reported during infection with *Schistosoma mansoni* in streptozotocin-induced diabetes in mice [30].

In a setting of diabetes, diabetic liver injury and non-alcoholic fatty liver disease contribute to lethal outcomes [31]. Although the pathogenesis and symptoms of opisthorchiasis are well established in otherwise healthy individuals [3, 4, 29], the morbidity due to co-occurrence of liver fluke infection and diabetes remains less well defined.

We investigated the pathogenesis of opisthorchiasis during diabetes using a hamster model. Disease features of diabetic liver studies in hamsters model the situation in humans, with well-known pathological changes, including steatosis, fibrosis, elevated levels of liver enzymes, necrosis of hepatocytes, biliary hyperplasia, up-regulated expression of TNF-α and NF-κB, oxidative stress, and liver dysfunction [18, 32]. We observed that cellular hypertrophy and hyperplasia of both hepatocytes and cholangiocytes together with accumulation of oxidative DNA damage, induction of inflammatory cytokines, and the dysregulation of cell proliferation-associated pathways and oxidative stress response intensified the hepatobiliary disease in diabetic hamsters infected with *O*. *viverrini*. Although some changes on hepatocytes might have been caused by the direct treatment with streptozotocin, rather than as the consequence of diabetes, the intensity of disease was nonetheless more severe in the liver fluke-infected, diabetic hamsters. The lesions reflected not only the diabetic condition but also the responses from the bile duct epithelia, hepatocytes, and sinusoids to antigens and metabolites from the liver fluke.

The phenotype of proliferation of biliary epithelia diverged among the treatment groups. In the diabetic hamsters, biliary hyperplasia localized to the lower order, smaller ducts whereas during liver fluke-infection, hyperplasia predominated in the higher order, larger diameter ducts, in agreement with earlier observations [10]. Proliferation of cholangiocytes that constitute the biliary epithelium is induced by cholestasis and biliary obstruction [33], leading to small bile duct proliferation and abnormalities of the bile canaliculi [34]. Similarly, proteins and other metabolites released by liver flukes, such as *O*. *viverrini* granulin, also induce bile duct proliferation, promote angiogenesis and wound healing, and impede apoptosis [35–38], all of which in turn are conducive to malignant transformation [39]. During infection with the related fluke *Clonorchis sinensis*, antigens diffuse through the biliary epithelium, provoking immunological reactions that damage hepatocytes [40]. The secreted antigen termed granulin from *C*. *sinensis* promotes progression and metastasis of CCA and hepatocellular carcinoma (HCC) [41]. In hamsters with concurrent opisthorchiasis and diabetes, additional, intense proliferation of cholangiocytes may follow from the enlargement of hepatocytes and pathophysiological alterations to the biliary canaliculi.

The synergistic damage due to both diabetes and opisthorchiasis was not confined to biliary epithelia; it was apparent also in hepatocytes, the major cell type of the liver parenchyma. The hepatocyte plays an essential role in diverse processes, and displays heroic regenerative capacity. Following injury and inhibition of proliferation of hepatocytes, two types of liver regeneration have been proposed. The first, termed compensatory hypertrophy, is characterized by increased hepatocyte cell volume. The second, termed stem/progenitor cell-mediated liver regeneration is self-explanatory. Onset of the latter follows on from the former [42]. Hepatocytes also have the potential to switch phenotypes, behaving like stem/progenitor cells [43]. Elevated levels of liver enzymes and conspicuous change in the gross appearance of the liver confirmed the hepatic injury in the OD hamsters. In addition, compensatory enlargement of hepatocytes increased intensively during joint diabetes and opisthorchiasis. We speculate that this phenomenon reflected proliferative responses by mature hepatocytes but, nonetheless, alone was insufficient to surmount the liver damage at large [43].

Immune responses to the liver fluke infection play a prominent role in the pathogenesis of opisthorchiasis [3, 4], and correspond to the time-course of liver fluke infection intensity and chronicity [22, 26]. After initiation of infection, *Il-12*-mediated Th1 cytokine responses, including *Il-1*, *Ifn-γ* and *Tnf-α* become apparent. Subsequently, expression of *Il-4*, *Il-10*, and *Tgf-β* reflected the acute phase of cell mediated the activation and the recruitment of cells of the adaptive immune response including fibroblasts. This progression in responses, in turn, drives pathogenesis including periductal fibrosis [11, 22, 26]. By contrast, hyperglycemia during diabetes contributes to susceptibility and persistence of infection [14]. Therefore, we investigated the tissue mRNA expression levels of informative cytokines to determine whether the immune response and function related to the severity of infection. Expression levels of *Tnf-α*, *Ifn-γ* and *Il-12*, *Il-4* and *Tgf-β* were significantly upregulated after infection, a cytokine milieu supportive of immunopathogenesis, which may damage the hepatobiliary tract. During the co-occurrence of opisthorchiasis and diabetes, *Il-6*, *Il-12* and *Il-13* were dominantly increased. Expression levels of the inhibitory cytokines *Il-10* and *Tgf-β* were generally unaffected during infection and/or diabetes, likely reflecting the impairment of immune response in diabetes [44] and participation of the cytokines in pathogenesis during liver fluke infection [22, 45]. Liver fibrosis is associated with upregulation of *Tgf-β* [46] and biliary periductal fibrosis and increased levels of *Il-6* characterize chronic opisthorchiasis [47]. In further agreement, both *Il-6* and *Il*-*13* were upregulated in OD hamsters compared with OV and DM. Overexpression of these cytokines during concurrent opisthorchiasis and diabetes may correlate with more severe disease of the hepatobiliary tract.

Diabetics face increased risk for CCA and HCC [48]. In northeastern Thailand, CCA and HCC are the most frequent cancers and the most frequent cause of death [49]. Inflammation-mediated oxidative stress, perhaps in tandem with other initiators that mutate chromosomal DNA is central to opisthorchiasis-induced CCA [29, 50]. Moreover, oxidative stress plays a prominent role in diabetes-associated liver injury, non-alcoholic steatohepatitis, and HCC [18, 51]. We observed significant changes in staining patterns of the oxidative stress marker, 8-oxo-dG, a molecular link from opisthorchiasis to opisthorchiasis-induced CCA [29]. Compared to other groups, the OD hamsters exhibited the most severe DNA damage and gross liver disease, the co-morbid sequelae of the involvement of infection-mediated oxidative stress superimposed on diabetic liver injury. We suggest that the intense oxidative DNA damage observed in the OD group could play a causative role in malignant transformation leading to CCA and to HCC.

The DNA polymerase co-factor PCNA plays a key role in diverse cellular activities [52]. Increased levels of PCNA reflect DNA replication and/or DNA repair, especially following DNA damage, occurring during both physiological and pathophysiological processes [53]. Enhanced PCNA activity was evident in both hepatocytes and cholangiocytes during infection and/or diabetes, particularly in the OD hamsters. This development suggested elevated levels of DNA repair and replication, and the exacerbation of levels of DNA damage and genotoxic lesions in this setting. The PI3K/AKT/mTOR pathway is essential to cellular metabolism and growth; pathway activation leads to cell proliferation, survival, and/or metabolic changes [54]. The DNA replication and/or DNA repair pathway is altered during disease such as with cardiovascular disease, diabetes and cancer including CCA [54–56]. The activation of this pathway is required for the protein phosphorylation by kinases. Impairment of the IRS/PI3K/AKT pathway was observed both during infection with *O*. *viverrini* and diabetes. Most proteins were upregulated during diabetes whereas most pathway members were down regulated during concurrent liver fluke infection and diabetes. These findings indicated that hepatocyte hypertrophy and biliary hyperplasia represent responses to cell damage during diabetes. In contrast, the co-occurrence of opisthorchiasis with diabetes might inhibit cellular proliferation due to the severe oxidative stress-induced DNA damage. The higher PCNA index reflected the greater level of DNA repair, with resultant increase in cell numbers and compensatory hypertrophy of liver tissue.

Hyperglycemia associates with cell proliferation both *in vivo* and *in vitro* such as in CCA cells [57, 58]. Glucose stimulated the proliferation both of cholangiocytes and hepatocytes in the hamsters and in H69 and HepG2 cells. The findings from hamsters highlighted the impact of opisthorchiasis and glucose levels on cell proliferation during early liver fluke infection, a phenotype that was not obtained in cultures H69s or HepG2s by the exposure to the secreted parasite antigens and metabolites (OVES) that we employed as a surrogate for infection. Infection with *O*. *viverrini* stimulated proliferation of hepatocytes, which decreased when exposed to OVES. By contrast, pulsing cholangiocytes with OVES retarded cell proliferation except at lower OVES concentrations that, by contrast, stimulated proliferation. The findings for cholangiocytes confirmed earlier reports [35] and the findings with H69 and HepG2 cells generally reflected the outcomes in the hamsters.

Nevertheless, one limitation of this study is that it employed the Syrian hamster infection model because this hamster is the most susceptible rodent to *O*. *viverrrini* infection [59]. Pathological changes in response to infection with *O*. *viverrrini* infection have been well described in hamsters [11] and the disease mimics human opisthorchiasis [8]. However, given that knock out hamsters and many immunological reagents including neutralizing antibodies against cytokines remain unavailable commercially for this rodent species, insight is limited with respect to mechanisms of the disease processes during the co-concurrence of opisthorchiasis and DM. Additionally, a type 2 DM-harboring hamster model (which remains unavailable) would likely be more appropriate for investigation of the effect of co-occurrence of type 2 DM and *O*. *viverrrini* on hepatobiliary pathogenesis, given the preponderance of Type 2 DM (rather than T1D) in liver fluke endemic regions [60].

**Fig 11:**
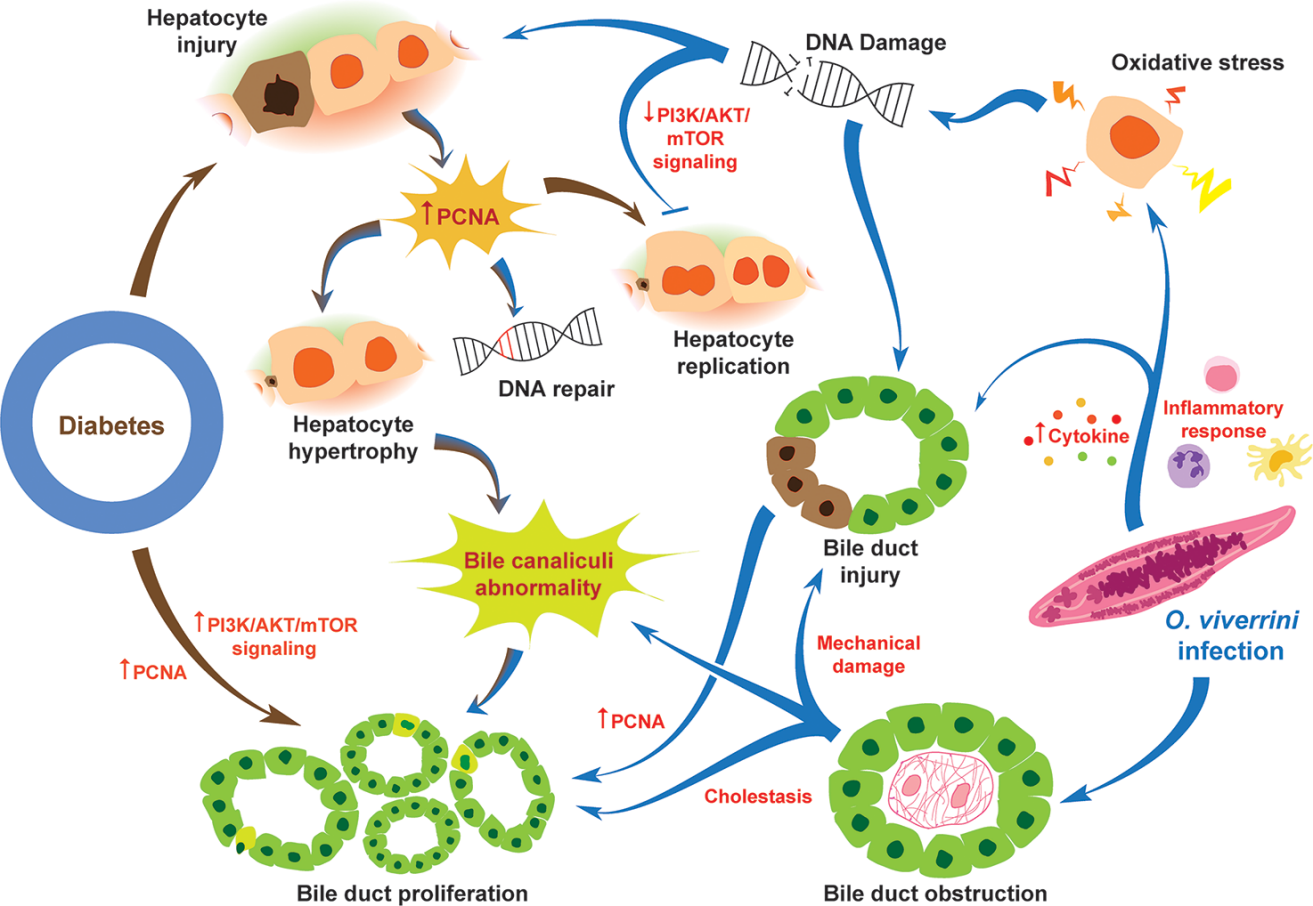
Schematic representation of processes hypothesized to exacerbate hepatobiliary tract disease during the co-occurrence of diabetes and opisthorchiasis due to infection with the food-borne liver fluke, *Opisthorchis viverrini*. During parasitism of the biliary tract by *O*. *viverrini*, oxidative processes damage the chromosomal DNA of hepatocytes and cholangiocytes, cholestasis follows from physical obstruction and blockage of biliary flow by worms within the lumen of bile ducts leading to proliferation of biliary epithelia and aberrant development of bile canaliculi (BC). Diabetes causes hepatocyte injury, contributes to hypertrophy of hepatocytes, to proliferation of bile ducts, and to defects in BC structure and development. Jointly, concurrent opisthorchiasis and diabetes exacerbate hepatobiliary disease.

Fig 11 summarizes our current interpretation of the pathways and connections that exacerbate morbidity during concurrent diabetes and opisthorchiasis: the combination of opisthorchiasis and diabetes aggravates liver fluke infection-induced hepatobiliary disease. During parasitism of the biliary tract by *O*. *viverrini*, oxidative processes damage the chromosomal DNA of hepatocytes and cholangiocytes, cholestasis follows from physical obstruction and blockage of biliary flow by worms within the lumen of bile ducts leading to proliferation of biliary epithelia and aberrant development of bile canaliculi (BC). Diabetes causes hepatocyte injury, contributes to hypertrophy of hepatocytes, to proliferation of bile ducts, and to defects in BC structure and development. Jointly, concurrent opisthorchiasis and diabetes exacerbate hepatobiliary disease, with multiplication of these problems evident in a spectrum of the pathophysiological sequelae including upregulation of expression of IL6 and other pro-inflammatory cytokines, oxidative DNA damage, and altered architecture of bile canaliculi, and hepatic hypertrophy and organomegaly. To conclude, in this hamster model, opisthorchiasis additively exacerbated diabetes-associated liver disease. The study provides insights for interventions for diabetes [15] during co-occurrence with opisthorchiasis, and likely other infectious agent-related hepatobiliary disease including CCA [61, 62].

## Methods

### Ethics statement

The Animal Ethics Committee of Khon Kaen University approved the study (AEKKU21/2556), based on the ethical guidelines for Animal Experimentation of the National Research Council of Thailand. Research involving hamsters was conducted in accordance with the guidelines for the Care and Use of Laboratory Animals of the National Research Council of Thailand.

### Induction of diabetes

**Fig 12:**
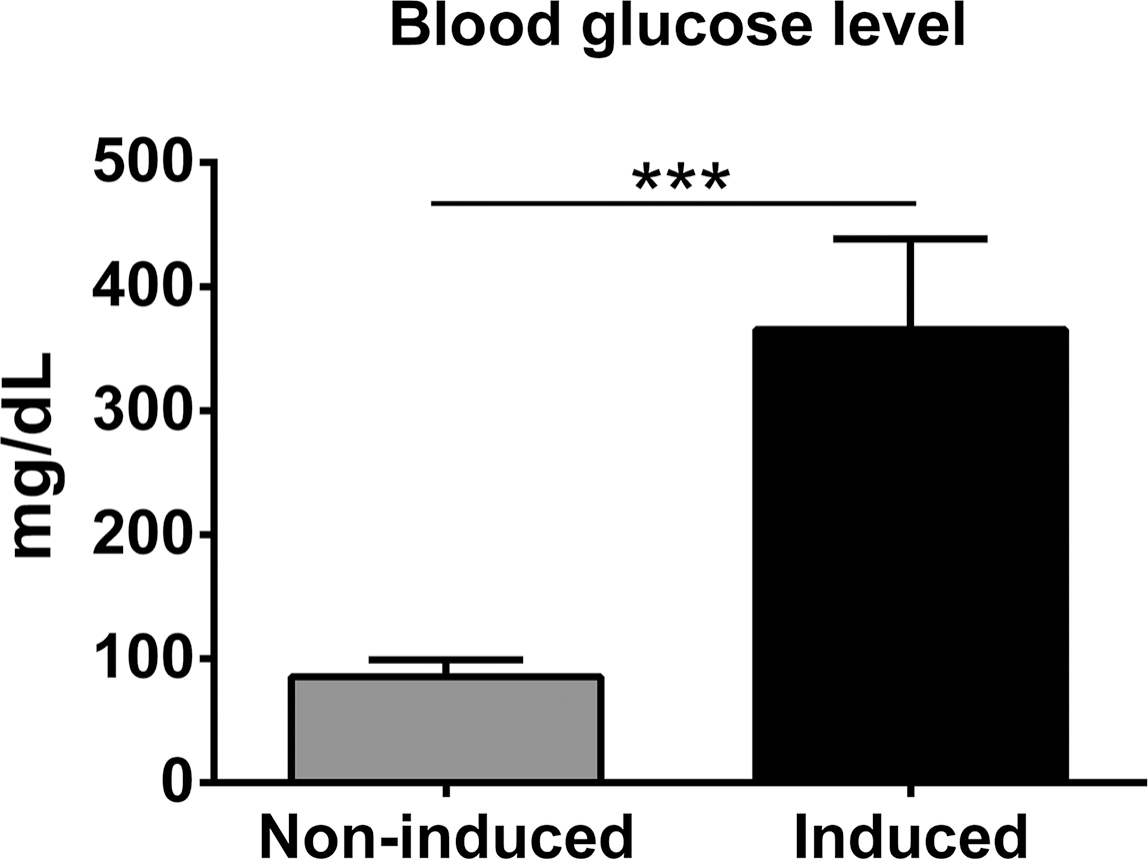
Levels of fasting blood glucose levels in hamsters at two weeks following induction of diabetes by streptozotocin. Fasting blood glucose level was estimated by glucometer in non-induced (n = 16, for normal and OV groups) and induced animal (n = 16 for DM and OD groups). Data are presented as mean ± S.D; ***P ≤ 0.001.

Thirty-two male 6- to 8-week-old Syrian golden hamsters, *Mesocricetus auratus*, average body weight 137 g, were obtained from the Animal Unit of Faculty of Medicine, Khon Kaen University. The rodents were maintained under a standard light cycle (12 hours dark-light cycles) and provided with *ad libitum* access to water and food (Smart heart, Thailand). General health was monitored daily and cage bedding changed twice a week. Sample sizes were calculated using the approach of Charan and Kantharia [63]. Eight hamsters were assigned to each of four groups: infection with *O*. *viverrini* (OV); induced diabetes (DM); infection and diabetes (OD); and control (N), i.e. uninfected and without diabetes. Type 1 diabetes was induced by intraperitoneal injection of 50 mg/kg body weight of streptozotocin (STZ) dissolved in 0.1 M tri-sodium citrate buffer, pH 4.5 (Sigma Chemical Co., St. Louis, MO) each day for three consecutive days [64]. Hamsters in the non-diabetes groups, i.e. Groups N and OV, received sodium citrate buffer alone. Diabetes was confirmed by glucometer (Accu-Chek Advantage II; Roche Diagnostics, Mannheim, Germany) to determine fasting blood glucose (FBG) at two weeks after STZ treatment. FBG ≥ 250 mg/dL indicated diabetes; hamsters with FBG ≥ 250 mg/dL were enrolled into the DM and OD groups (Fig 12).

### *Opisthorchis viverrini*: Infection, collection of adult worms, antigen preparation

Metacercariae of *O*. *viverrini* were obtained from naturally infected cyprinid fishes [65], and examined by light microscopy to observe larval movement within the cyst before inclusion in the inoculum. Metacercariae that did not exhibit larval movements were discarded. Hamsters were infected by stomach intubation with 50 viable metacercariae [10, 45]. Hamsters in the OD group were infected with the metacercariae after occurrence of diabetes (FBG ≥ 250 mg/dL).

Infection of hamsters and other manipulations including phlebotomy were performed under ether anesthesia, with concerted effort made to minimize pain and suffering to the hamsters. Hamsters were euthanized with an overdose of inhaled ether. At necropsy at eight to 16 weeks after liver fluke infection, adult *O*. *viverrini* worms were recovered from the liver as described [10]. At one month following infection with *O*. *viverrini*, the hamsters were starved for two days, weighed and euthanized by inhalation of over dose of diethyl ether. Blood/serum, liver, and worms were collected via cardiac puncture. Wet weight of the liver was measured, after which livers were sliced by scalpel into three similar sized fragments: one fragment was snap frozen in liquid nitrogen, the second stored in Trizol reagent (Ambion, Thermo Fisher Scientific, Walthem, MA), and the third fixed in 10% buffered formalin, with the first and second fragments stored at -80°C.

Excretory/secretory products of adult *O*. *viverrini* liver flukes in vitro were prepared as described [11, 66]; here these excretory/secretory products are termed OVES. Subsequently, extraction of OVES was subjected to Triton-X114 (Sigma) extraction followed by incubation with Bio-Beads SM2 (Bio-Rad Laboratories, Hercules, CA) was undertaken to remove residual bacterial lipopolysaccharide (LPS). Protein concentration of OVES was determined by the Bradford assay, after which aliquots of OVES were stored at -80°C.

### Liver function enzymes

Levels of the diagnostic liver function enzymes, aspartate transaminase (AST), alanine transaminase (ALT), and alkaline phosphatase (ALP) in sera of hamsters were ascertained using reagents from Thermo Trace (Melbourne, Australia) and an automated spectrophotometer (Technicon RA100, Luton, UK).

### Histological and immunohistochemical analysis

Thin sections of liver embedded in paraffin were cut using a microtome, at a thickness of 5 μm, and stained with hematoxylin and eosin (H&E). For immunohistochemical staining, antigens were retrieved following deparaffinization and rehydration, and the sections probed with primary and secondary antibodies (Table 2), as described [11]. For immunoperoxidase staining, the signal was detected using diaminobenzidine (DAB) as the substrate, followed by counterstaining with hematoxylin. For immunofluorescence, slides were counter-stained with Hoechst 33342 dye (Thermo Fisher Scientific). Stained slides were examined using a Nikon Eclipse Ni-U light microscope fitted with a Nikon ES-Ri1 digital video camera driven by NIS-Element D 4.00 software (Nikon instrument Inc., Melville, NY).

**Table 2:** List of antibodies and assay dilutions.

Antibody	Dilution	Host	Vendor
**Immunohistochemistry**			
**Immunoperoxidase**			
*Primary antibody*			
anti-Proliferating cell nuclear antigen (anti-PCNA)	1: 1000	Rabbit	Abcam
CD-10	1: 50	Rabbit	Santa Cruz Biotechnology
Soluble extract of adult *O*. *viverrini* worms	1: 100	Rabbit	[11]
*Secondary antibody*			
Rabbit-IgG		Goat	Jackson ImmunoResearch
**Immunofluorescence**			
*Primary antibody*			
8-oxodG	1: 100	Mouse	JAICA
*Secondary antibody*			
Alexa Fluoro 488-labeled anti-mouse IgG	1: 400	Goat	Invitrogen
**Western blot**			
*Primary antibody*			
AKT	1: 1000	Rabbit	Cell Signaling Technology
Phospho-AKT (ser473)	1: 2000	Rabbit	
Phospho-AKT (Thr308)	1: 1000	Rabbit	
PI3 Kinase p85	1: 1000	Rabbit	
PI3 Kinase p110α	1: 1000	Rabbit	
Phospho-PI3 Kinase p85 (Tyr485)/p55, (Tyr199)	1: 1000	Rabbit	
PTEN	1: 1000	Rabbit	
Phospho-PTEN (Ser380/Thr382/383)	1: 1000	Rabbit	
mTOR	1: 1000	Rabbit	
Phospho-mTOR (Ser2448)	1: 1000	Rabbit	
β-actin	1: 2000	Mouse	Abcam
Insulin receptor beta	1: 1000	Mouse	
IRS1	1: 300	Rabbit	Santa Cruz Biotechnology
*Secondary antibody*			
Rabbit IgG		Goat	Jackson ImmunoResearch
Mouse IgG		Sheep	

Ten randomly selected, H&E stained regions in each microscopic field, were counted under high magnification, to ascertain cell numbers of hepatocytes. The findings are presented as numbers of cells per field. Two independent investigators each counted 10 regions; and the findings were pooled. PCNA-positive stained nuclei were counted at x200 magnification from 10 fields in each case, to establish a DNA synthesis/repair index for hepatocytes [67]. The mean number of DNA synthesis/repair index of each case was calculated as follows: number of positive nuclei/total number of countered stained x 100. In biliary periductal sites, PCNA-positive nuclei were semi-quantitatively graded as follows; negative, number of positive biliary cells was less than 1% of total biliary cells; 1+, 1–25% of total biliary cells; 2+, 25–50%; 3+, > 50% [10]. Nuclei positive for 8-oxo-dG were counted at x400 magnification (three fields selected at random) [68]. To determine the structure of bile canaliculi, the patterns of CD10 staining [69] were graded based on the percent of positive cells from 10 areas examined in each sample at a magnification of x400, as follows: negative, zero CD10-positive; 1+, <10% of CD10-positive areas; 2+, 10–50% of CD10-positive areas; 3+, >50% of CD10-positive areas [9]. Periductal fibrosis was graded under bright-field microscope into five stages: grade 0 = no fibrosis; grade 1 = mild fibrous expansion of some portal areas; grade 2 = moderate fibrous expansion of most portal areas with short fibrous septa; grade 3 = severe fibrous expansion of most portal areas with occasional portal to portal bridging; and grade 4 = more severe fibrous expansion of most portal areas with marked bridging [26].

### TUNEL assay

DNA fragmentation, an apoptotic marker, within cells fixed in the thin sections of liver was detected *in situ* (TACS•XL DAB *In Situ* Apoptosis Detection Kit, Trevigen Inc., Gaithersburg, MD). Terminal deoxynucleotidyl transferase (TdT) dUTP nick-end labeling (TUNEL)-positive cells were counted under high power microscopy on 10 non-overlapping areas in each case and are presented as percent positive cells per total number of cells counted in the field [70].

### Western blotting

Twenty to 40 μg of protein extracted from hamster liver separated by sodium dodecyl sulfate polyacrylamide gel electrophoresis (SDS-PAGE), 20% separating gel, 5% stacking gel), was transferred to a polyvinylidene difluoride (PVDF) membrane (Amersham Bioscience, GE Healthcare Bio-Sciences, Marlborough, MA). Protein-binding sites on membranes were blocked by incubation in 5% skimmed milk powder in 1x phosphate-buffered saline (PBS)/0.05% Tween-20 (PBS-T) for 2 hours at 23°C. Thereafter, membranes were probed sequentially with primary antibodies (S2 Table) and horseradish peroxidase-conjugated secondary antibody. Reactive bands were detected using chemiluminescence (ECL Plus, GE Healthcare). The intensity of reaction bands was quantified using the ImageJ open platform, https://imagej.nih.gov/ij/plugins/ (NIH, Bethesda, MD).

### Quantitative reverse transcription polymerase chain reaction

Total RNA was extracted from hamster livers using the Trizol or RNAzol systems. Quantity and quality of RNA was assessed by spectrophotometry, after which the RNA was reverse transcribed to complementary DNA (cDNA) using the iScript cDNA system (Bio-Rad, Hercules, CA). Quantitative real time PCR was accomplished using primers specific (Table 3) and the LightCycler 480 SYBR green I master (Roche Applied Science, Mannheim, Germany) or Applied Biosystems 7500 Real-Time PCR System (Thermo Fisher Scientific). The comparative C_T_ method, $2^{-\Delta \Delta C_{T}}$ [71] was used to determine the relative mRNA expression level using hypoxanthine phosphoribosyltransferase 1 (*hprt1*) as the internal standard.

**Table 3:** Target genes, GenBank accessions, and primer oligonucleotides.

Hamster gene	Accession number	Oligonucleotide primer
*Tnf-α*	AF046215.1	F: 5’-GACGGGCTGTACCTGGTTTA-3’
		R: 5’-GAGTCGGTCACCTTTCTCCA-3’
*Ifn-γ*	NM_001281631.1	F: 5’-TATCTTGACGAACTGGCAAA-3’
		R: 5’-AACCTGAAGGTCATTTACCG-3’
*Il-6*	AB028635.1	F: 5’-GACTTCACAGAGGACACTAC-3’
		R: 5’-CACATAGTCATTGTCCATACAG-3’
*Il-12*	NM_001281689.1	F: 5’-GGCCCAGCACAAGTATAAAT-3’
		R: 5’-CTTTCCTTTCTCTCTTGCGA-3’
*Il-4*	AF046213.1	F: 5’-GCCATCCTGCTCTGCCTTC-3’
		R: 5’-TCCGTGGAGTTCTTCCTTGC-3’
*Il-13*	XM_005067910.2	F: 5’-CGTCGCCTGCCTTGGTGG-3’
		R: 5’-CAATATCCTCTGGGTCTTGT-3’
*Il-10*	AF046210.1	F: 5’-AAGGGTTACTTGGGTTGC-3’
		R: 5’-GGAGAAATCGATGACAGC-3’
*Tgf-β*	AF046214.1	F: 5’-ACATCGACTTTCGCAAGGAC-3’
		R: 5’-TGGTTGTAGAGGGCAGGAC-3’
*Hprt1*	DQ403055.1	F: 5’-AGTCCCAGCGTCGTGATTAG-3’
		R: 5’-CAGAGGGCCACAATGTGATG-3’

### H69 cholangiocytes and HepG2 hepatocarcinoma cells

The immortalized human cholangiocyte H69 cell line was maintained in DMEM/F-12 (Gibco, USA) high glucose, growth factor supplemented media [72]. HepG2 cells (ATCC, Manassas, VA) were maintained according to ATCC’s protocol. Conditioned media for H69 and HepG2 were prepared as described [58, 73]. Glucose at 1M was included to achieve target concentration of glucose in the H69 medium. To avoid hyperosmolarity, an equivalent concentration of mannitol at 1 M was included in the medium of control groups. For HepG2, a mixture of DMEM low glucose and DMEM high glucose media (Thermo Fisher Scientific) served as the conditioned medium.

### Real time assessment by xCELLigence system of cell proliferation

Cell growth was evaluated using the xCELLigence DP system (ACEA Biosciences, San Diego, CA), designed to monitor events in real time by measuring electrical impedance across interdigitated microelectrodes integrated on the bottom of tissue culture E-plates (ACEA), see http://www.aceabio.com/main.aspx [35, 74]. H69 and HepG2 were seeded at 5,000 and 10,000 cells/well, respectively, into E-plates in medium supplemented with 10% fetal bovine serum (FBS), and cultured in a humidified incubator at 37°C, 5% CO_2_ for one day. The cells were rinsed in PBS and medium replaced with H69 or HepG2 medium diluted 1 in 20, i.e. 0.5% FBS, for four to six hours. Thereafter, the medium was replaced with conditioned medium and the cells monitored continuously, at intervals of 15 minutes, for up to 120 hours using the RTCA Software 1.2 (ACEA), as described [37, 39].

### Statistical analysis

Graded scores of the stained tissue sections were compared using the non-parametric Mann-Whitney U test, using IBM SPSS statistics package, version 19 for Windows and version 20 for Mac (IBM corporation, NY). With xCELLigence data, normalized cell index (CI) curves with two-way ANOVA were generated and analyzed using GraphPad Prism 7 (GraphPad Software, La Jolla, CA). Findings are presented as means ± S.D. Statistical differences among groups were assessed using Analysis of Variance (ANOVA), Tukey’s honest significance test, and Student’s *t*-test. *P*-values of ≤ 0.05 were considered to be statistically significant.
